# Probiotics Reduce Postoperative Infections in Patients Undergoing Colorectal Surgery: A Systematic Review and Meta-Analysis

**DOI:** 10.1155/2017/6029075

**Published:** 2017-04-06

**Authors:** Peng Cheng Liu, Yu Ke Yan, Yu Jing Ma, Xiang Wen Wang, Jie Geng, Man Cai Wang, Feng Xian Wei, Ya Wu Zhang, Xiao Dong Xu, You Cheng Zhang

**Affiliations:** ^1^Department of General Surgery, Lanzhou University Second Hospital, Lanzhou 730030, China; ^2^Hepato-Biliary-Pancreatic Institute, Lanzhou University Second Hospital, Lanzhou 730030, China

## Abstract

*Background.* We performed this meta-analysis to investigate the efficacy of probiotics on prevention of infection-related complications following colorectal resection. *Method.* PubMed, EMBASE, Cochrane Library, and the Web of Science were searched up to January 2016. According to the results, only randomized controlled trials that compared the efficacy of probiotics on patients with colorectal resection were included for meta-analysis. *Results.* Nine studies including a total of 1146 patients met the criteria (556 received multistrain probiotic bacteria, 590 with non-multistrain probiotic bacteria). The combination of multistrain probiotics was beneficial in the reduction of total infections (OR = 0.30, 95%CI: 0.15–0.61, *p* = 0.0009), including surgical site infections (SSI) (OR = 0.48, 95%CI: 0.25–0.89, *p* = 0.02) and nonsurgical site infections (NSSI) (OR = 0.36, 95%CI: 0.23–0.56, *p* < 0.00001). However, there was no significant reduction in total infections (OR = 0.74, 95%CI: 0.50–1.09, *p* = 0.13) or SSI (OR = 0.77, 95%CI: 0.52–1.12, *p* = 0.17) with the application of non-multistrains of probiotics. *Conclusion.* Combinations of multistrain probiotic bacteria showed promise in preventing the incidence of infections following colorectal surgery. However, the efficacy of one or two strains of probiotics remains undetermined.

## 1. Introduction

Although surgical techniques and perioperative care have been greatly improved, postoperative infection still remains a major complication that prolongs hospitalization and increases costs, especially after colorectal procedure [1, 2]. Reasons for infectious complications may be related to the stress of operation, damage of intestinal mucosa, imbalance of intestinal flora, dysfunction of the local immune system, and the transfer of bacteria [3]. Although intravenous administration of antibiotics has proven effective on preventing infections, antimicrobial resistance raises a common concern. In addition, antibiotics may aggravate the disturbed flora associated with infectious complications. New treatment or prevention strategies are urgently needed.

Probiotics are live microorganisms, which are called synbiotics when used in combination with prebiotics (nondigestible food constituents). Pro-/synbiotics are known for their beneficial effects on maintenance of normal enteric flora and intestinal barrier function, regulation of gut immune function, inhibiting colonization of pathogenic strains, and providing health benefits to the host [4–9]. Some researchers have concluded that gut microbiota dysbiosis occured in many medical conditions, such as diarrhea [10], cholesterol gallstones [11], liver cirrhosis [12, 13], and Crohn's disease [14]. In recent years, a number of clinical trials have also illustrated a decrease in the incidence of postoperative infectious complications when probiotics were used in patients with hepatectomy, pancreaticoduodenectomy, and liver transplants [15–17]. Furthermore, some studies indicate that probiotics are able to prevent the occurrence of tumor, of course including colorectal cancer [18, 19]. Likely, there are attractive prospects for probiotics in colorectal surgery.

Although several randomized controlled trials (RCTs), which focus on the use of probiotics in prevention of postoperative infections, were performed in patients who underwent colorectal resection, only a few of them found that probiotics can improve the integrity of the gut mucosal barrier and then decrease infectious complications [20–22]. Interestingly, some researchers reported that probiotics administration did not reduce the postoperative inflammatory response and prevent infection [23–27]. Differences in study design and sample size are suspected to be the main reason for these controversy conclusions. Therefore, a meta-analysis, which can pool data from existing RCTs together and assess the clinical efficacy of probiotics on postoperative infections, is necessary. In this paper, we review results from relevant high-quality literature to determine whether perioperative probiotic treatment can reduce infection-related complications in elective colorectal surgery or not.

## 2. Methods

### 2.1. Inclusion and Exclusion Criteria

All published RCTs that assessed the efficacy of probiotics for colorectal surgery infections and complications were included in this meta-analysis. The types, formula, dose, and durations of the probiotics were not limited because there was no data to assume an optimal prophylactic treatment. Conversely, we excluded studies based on the following criteria: (1) patients who received preoperative chemoradiotherapy; (2) only prebiotics were administered during the perioperation period; (3) lack of key data; and (4) duplicate studies published by the same intuitions (in which case, we selected the highest quality and the latest publications, unless endpoints were mutually exclusive).

### 2.2. Literature Search

We conducted our search in four databases: PubMed (January 1966 to January 2016), EMBASE (January 1990 to January 2016), Cochrane Library (Issue 1, 2016), and the Web of Science (January 1985 to January 2016). Key searching terms included “*probiotic*,” “*synbiotic*,” “*lactobacillu*s,” “*bifidobacterium*,” “*colorectal surgery*,” “*colon cancer*,” and “*rectal cancer*.” In addition, general review articles and references were also scrutinized for additional eligible studies. According to the inclusion criteria, two reviewers independently searched the four databases and reviewed each study. Trials with repeated titles, irrelevant, poor quality, or little information were excluded. Disagreements between the two reviewers were resolved through consultation with Y.C. Zhang.

### 2.3. Data Extraction

Two reviewers independently extracted data by using prespecified data collection forms. Discrepancies in data extraction were resolved through discussion and consultation with the senior investigator (Y.C. Zhang) when necessary. Relevant information extracted from each eligible study included the name of the first author, publication data, study design/setting, patient characteristics, existence of any types of infectious complications, and the types, dosage, and treatment durations of probiotics. The extracted data were crossed check by another reviewer independently.

### 2.4. Endpoints and Criteria for Analysis

This study was concerned about the following outcomes: total infections, surgical site infection (SSI), nonsurgical site infection (NSSI), bacterial translocation, and anastomotic leakage. Total infections was defined as any infection that occurred during hospitalization. SSI included incision infection and organ/space SSI; the former means that the infection is present in the surgical wound and the latter is infection specific to the surgical region. NSSI included urinary tract infections, pneumonia, and bacteremia. All of which were assessed by imaging, laboratory examinations, or a positive bacterial culture. Bacterial translocation (BT) was determined using a culture of mesenteric lymph nodes that was obtained following examination of the abdomen and bowel mobilization. Anastomotic leakage indicated suture failure and discharge of intestinal content from drainage tubes.

### 2.5. Assessment of Methodological Quality

The quality of the studies included in meta-analysis was assessed by two reviewers independently. Not only the Cochrane Risk of Bias Tool for RCTs was adopted [28] but we also followed Jadad criteria (maximum score 5) guidelines. A trial with a score of 3 or more was regarded as high quality [29]. Any differences were resolved through discussion with the senior investigator.

### 2.6. Statistical Analysis

Data were pooled using Review Manager Software (version 5.2). For dichotomous outcomes, we used odds risk (OR) with 95% confidence intervals (CIs). *p* values less than 0.05 were considered an indication of statistical significance. The chi-square test (*χ*^2^) was performed to assess the statistical heterogeneity among studies, and *I*^2^ value was used to assess the extent of inconsistency [30]. Generally, *χ*^2^ test with a *p* value < 0.10 indicated significant heterogeneity across studies. When *I*^2^ value was 50% or greater, a random effect model was used. Conversely, if *I*^2^ was less than 50%, the fixed effect of meta-analysis was applied. Subgroup analyses were performed to identify the effects of different probiotic formulations. Finally, overall effects were analyzed by performing a *Z*-test. The present study has been performed and complied with the PRISMA guidelines [31].

## 3. Results

### 3.1. Literature Search Results

**Figure 1:**
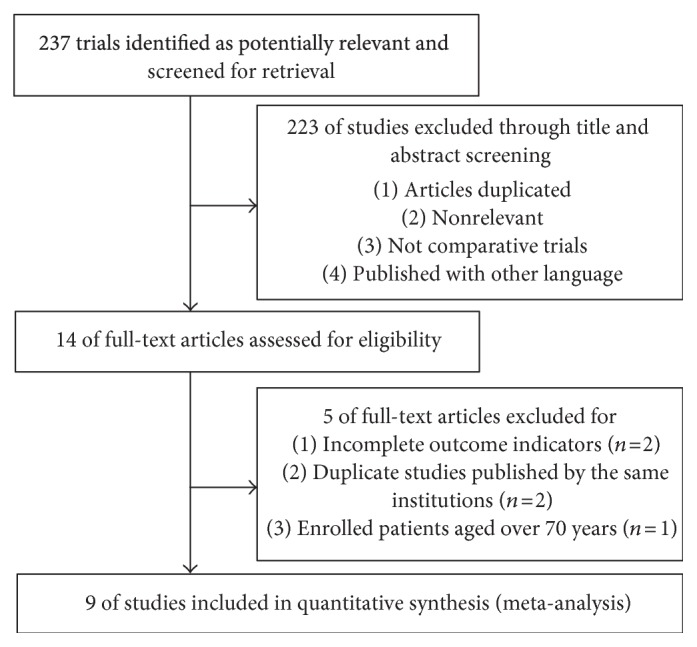
Flow chart of study search. A total of 9 studies ultimately included for meta-analysis.

We identified 237 potentially relevant studies from the databases, and of those, 14 full texts based on the title and abstract reviews were selected (Figure 1). During subsequent reviews of the full texts, 2 studies were excluded because outcome indicators were incomplete, 2 studies were excluded due to report duplication, and 1 study was excluded because it enrolled patients all aged over 70 years. The remaining 9 studies were included for quantitative synthesis and meta-analysis [21–28, 32].

### 3.2. Study Characteristics

All included trials were randomized and were either double-blind or single-blind studies.  Table 1 lists the characteristics of the included studies. There were a total of 1146 participants in the 9 studies. 562 patients were in experimental groups with probiotics administration, and 584 patients were in control groups without. Six studies used a combination of multistrain probiotic bacteria [20–22, 24, 26, 27, 32]. Three of these used 4 types of probiotic bacteria in combination [20, 26, 27], while three used 3 types [21, 22, 32]. In addition, one trial utilized a double probiotic agent [24], and two used only single strains of probiotic bacteria [23, 25]. Another seven trials used lactobacilli and bifidobacterium, while three used streptococcus. Lastly, only one trial used pediococcus and enterococcus (see Table 1).

### 3.3. Methodological Quality

**Figure 2:**
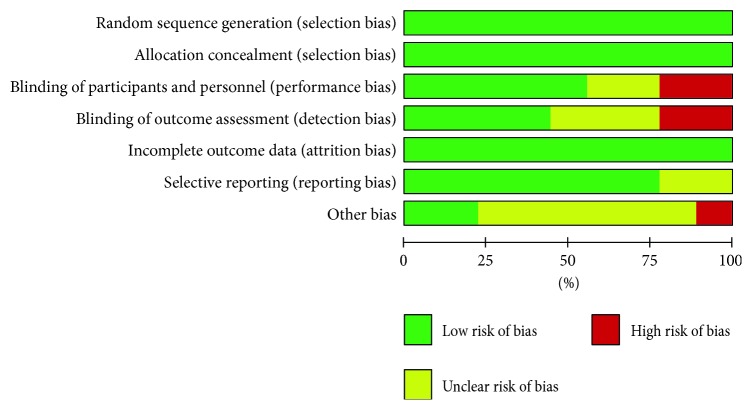
The results of the bias risk assessments.

Overall, the risk of bias was low (Figure 2). Patients analyzed in the studies were adequately randomly divided into two groups by using computer-generated random numbers or by random number sequence [20–27]. Treatment allocation concealment by sealed opaque envelopes was implemented in four studies [20, 26, 27, 32], and physicians were blinded to treatment options in five [21, 22, 26, 27, 32]. None of the studies reported an outcome of incomplete data. Additionally, all studies achieved a Jadad score of 3 or more (Table 1).

### 3.4. Total Effects of the Probiotics

Types and formulas of the probiotics were ignored when determining the total effects of probiotics. Five of the nine studies reported total infections [20, 22–25]. There was no evidence of heterogeneity between the trials (*I*^2^ < 50%, *p* = 0.13) and the fixed model that was applied. Here, evidence of probiotics administration reducing total infections was gotten [OR = 0.59, 95%CI (0.43, 0.83), *p* = 0.002] as shown in Table 2.

For surgical site infection, 7 trials reported incision infection [20, 22, 24–27, 32] and 5 reported organ/space SSI [23–25, 27]. Pooled results suggest that probiotics were beneficial in the reduction of SSI complications [OR = 0.67, 95%CI (0.49, 0.93), *p* = 0.02]. Results of subgroup analysis showed evidence of probiotics administration reducing incision infection [OR = 0.61, 95%CI (0.41, 0.91), *p* = 0.02] (Table 2), and there was no heterogeneity between studies (*I*^2^ = 0%, *p* = 0.60). However, to organ/space SSI, fixed model was applied (*I*^2^ = 0%, *p* = 0.53), and there was no difference between the two groups [OR = 0.82, 95%CI (0.47, 1.42), *p* = 0.48] (Table 2).

In our study, nonsurgical site infections included urinary tract infections, pneumonia, and bacteremia. Of the nine studies in the meta-analysis, three reported urinary tract infections [20, 21, 23], and four reported pneumonia and bacteremia [20–22, 28]. Probiotics were shown to reduce urinary tract infections [OR = 0.39, 95%CI (0.16, 0.96), *p* = 0.04], pneumonia [OR = 0.25, 95%CI (0.11, 0.60), *p* = 0.002], and bacteremia [OR = 0.44, 95%CI (0.23, 0.85), *p* = 0.01] (Table 2). There was no evidence of heterogeneity in urinary tract infections (*I*^2^ = 3%, *p* = 0.36), pneumonia (*I*^2^ = 0%, *p* = 0.92), or bacteremia (*I*^2^ = 0%, *p* = 0.46). Then we pooled estimate of nonsurgical site infections, and the results indicated that probiotics were indeed beneficial in the reduction of nonsurgical site infections [OR = 0.36, 95%CI (0.23, 0.57), *p* < 0.00001] (Table 2).

Next, we evaluated the effect of probiotics in bacterial translocation and anastomotic leakage. Two of the nine studies reported bacterial translocation [21, 27], and four reported anastomotic leakage [20, 22, 24, 25]. The differences between the probiotics group and the control group were not statistically significant in neither bacterial translocation [OR = 0.13, 95%CI (0.01, 1.48), *p* = 0.10] nor anastomotic leakage [OR = 0.80, 95%CI (0.28, 2.48), *p* = 0.70]. A random effect model was used because the statistical heterogeneity was significant in both bacterial translocation (*I*^2^ = 84%, *p* = 0.01) and anastomotic leakage (*I*^2^ = 58%, *p* = 0.07) (Table 2).

### 3.5. Efficacy of Different Probiotic Formulations

We analyzed the effects that different formulations of probiotics had on postoperative infections. Six of the nine studies administered with multistrain probiotics [20–22, 26, 27, 32], three of which involved 4 types of probiotic bacteria combination [20, 26, 27] and another three used 3 types of probiotic bacteria combination [21, 22, 32]. Evidence indicates that multistrain combinations have a positive effective on total infections [OR = 0.30, 95%CI (0.15, 0.61), *p* = 0.0009], incision infections [OR = 0.38, 95%CI (0.19, 0.76), *p* = 0.006], urinary tract infections [OR = 0.34, 95%CI (0.13, 0.90), *p* = 0.03], pneumonia [OR = 0.27, 95%CI (0.11, 0.63), *p* = 0.003], and bacteremia [OR = 0.44, 95%CI (0.23, 0.85), *p* = 0.01]. Then, we pooled estimate of multistrain combinations for SSI and NSSI, which indicated that multistrain probiotic combinations were beneficial in the reduction of SSI [OR = 0.48, 95%CI (0.25, 0.89), *p* = 0.02] and NSSI [OR = 0.36, 95%CI (0.23, 0.56), *p* < 0.00001], as shown in Table 3.

One of the trials [24] used a double probiotic agent, and the other two used single-strain probiotic bacteria [23, 25]. These three trials were not considered multistrain combinations. The effect of these probiotics on postoperative infection was investigated, and the results showed no risk reduction in total infections [OR = 0.74, 95%CI (0.50, 1.09), *p* = 0.13], incision infections [OR = 0.79, 95%CI (0.48, 1.31), *p* = 0.36], organ/space SSI [OR = 0.73, 95%CI (0.41, 1.31), *p* = 0.29], or anastomotic leakage [OR = 1.49, 95%CI (0.78, 2.86), *p* = 0.23]. Furthermore, no heterogeneity was recognized (*I*^2^ < 50%). Lastly, we pooled estimate of these combinations for SSI and yielded no evidence of one or two strains of probiotics being beneficial in the reduction of postoperative SSI [OR = 0.77, 95% CI (0.52, 1.12), *p* = 0.17] (Table 3).

## 4. Discussion

Probiotics was described as live microorganisms, which has beneficial effects to host by maintaining gut microbial balance and improving the local immune system [8, 9]. A number of RCTs and meta-analyses have documented that probiotics have positive alternative for some disease, such as cirrhosis [33], antibiotic-associated diarrhea [34], hepatectomy [16, 35], and pancreaticoduodenectomy [17]. However, no therapeutic or preventive effects were shown by probiotics for ulcerative colitis [36], Crohn's disease recurrence [37], and severe acute pancreatitis (SAP) [38]. A review based on several studies concluded that probiotics in colorectal surgery does not influence on the incidence of postoperative infections [39]. He et al. [40] conducted a meta-analysis of six RCTs with 361 patients and concluded that administration of perioperative probiotics does not reduce the incidence of complications such as incision infection, anastomotic leak, and bacteremia.

In our meta-analysis, we showed that probiotics, especially combinations of multistrains (at least 3), were effective in preventing total infections after colorectal surgery, including SSI and NSSI. These results are contrary to a research by He et al. [40]. Small sample size and significant heterogeneity in He's study might have influenced the reliability and validity of the conclusions. In contrast, the present meta-analysis included 1146 participants from nine high-quality studies (Jadad scores are 3 or more, as displayed in Table 1). We also evaluated bacterial translocation (BT), which means that viable bacteria is translocated from intestine to intestinal mesenteric lymph nodes or distant organs through an impaired intestinal mucosal barrier [21]. Interestingly, our meta-analysis indicated that multiple combinations of probiotics could diminish BT and anastomotic leakage, while non-multiple-strain administration cannot. Liu et al. found that the prophylactic probiotics administration for postoperative patients with colorectal cancer could improve the integrity of gut mucosal barrier by benefiting the fecal microbiota and enhancing the mucosal tight junction protein expression [32]. In a recent study [20], Kotzampassi supported that the benefit action of probiotics for colorectal surgery may be related either with the earlier bowel movement or with modulation of the innate immune responses. The same authors conducted another trial that used probiotics for multidrug-resistant (MDR) *Pseudomonas aeruginosa* infection in mice. The results indicated that pretreatment with *Lactobacillus plantarum* significantly prolonged survival, and the intracellular mechanism may be related to suppress the expression of *SOCS3* (suppressor of cytokine stimulation-3) and increase the production of TNF-*α* and IL-10 [41]. Therefore, possible explanations for the effects of probiotics may be contributed to the improvement of the intestinal barrier, inhibition of pathogens, recovery of intestinal peristalsis, and/or enhancement of immune responses [42].

However, it should be noted that the formula, dose, and treatment duration of the probiotics vary considerably between studies, because there was no data to establish an optimal prophylactic treatment. We analyzed the different formulas of probiotics for postoperative infection; the results indicated that a combination of multistrain bacteria (at least three) has the significant effect on total infections, SSI, and NSSI after colorectal surgery. For the above results, the potential mechanisms probably include increasing a diversity of the intestinal microbiota, acting a synergetic effect, and offering a healthy normal microbiota by multistrain probiotics. However, all types of regimens may not be truly equivalent. A systematic review analyzed 72 articles and concluded that some probiotic products, particularly *Lactobacillus rhamnosus* GG or *Saccharomyces boulardii*, increased the risk of complications in patients with organ disorders. Administration with these probiotics could result in bacteremia or fungemia in patients [43]. Therefore, different probiotics and formulations may function differently in various clinic situations. Despite no apparent adverse reactions were observed in any of the studies included in this meta-analysis, routine surveillance for side effects during administration is still needed.

Regarding dosage, the optimal doses for specific diseases are not well established. In general, commercially available probiotic formulas generally contain ≥10^6^ CFU of viable organisms. One meta-analysis indicated that *Lactobacillus* GG was the most effective in treating acute gastroenteritis when used with a daily dose ≥10^10^ CFU [44]. In our meta-analysis, probiotic dosage varied greatly among the 9 RCTs. Liu et al. adopted a mixture of 3 probiotic bacteria in one group: *Lactobacillus plantarum* (cell count ≥ 10^11^ CFU/g), *Lactobacillus acidophilus*-11 (cell count ≥ 7.0 × 10^10^ CFU/g), and *Bifidobacterium longum*-88 (cell count ≥ 5.0 × 10^10^ CFU/g) [21]. Patients in the control group received encapsulated maltodextrin daily. Results indicated lower infection rate in the probiotics group, and during the postoperative 72 h period, the total rate of positive bacterial cultures (including blood, central lines, and sputum) was significantly higher in the control group. In another study [22], the patients in the probiotic group received triple probiotics containing 10^8^ CFU/g of *B*. *longum*, *L*. *acidophilus*, and *Enterococcus faecalis*, and it was concluded that triple probiotics decreased postoperative infection complications (10% versus 33.3%). However, a recent study that compared administration of *Saccharomyces boulardii* (containing 5 × 10^8^ CFU/g) perioperatively with placebo showed no significant difference in postoperative infections [23]. These conflicting results may be due to the difference in bacterial species, formula, and the number of probiotics used.

Presently, it is not clear that whether the effects of probiotics are influenced by the duration of the treatment. Gou et al. found that if treatment duration was 15 days or less, there were significant improvements in almost all outcomes of severe acute pancreatitis and critical illness [38]. In our meta-analysis, subgroup analyses were also performed based on the treatment duration that was more than 10 days or less, and both of subgroups showed significant improvement in total infections (see Figure S1 in Supplementary Material available online at https://doi.org/10.1155/2017/6029075, published online). However, only the group with treatment duration of more than 10 days showed the significant efficacy for incision infection (see Figure S2, published online). Nevertheless, since there were only a small number of participants in these studies, no reliable conclusions regarding treatment duration can be made.

Finally, probiotics did not reduce infection-related complications in 3 of the studies reviewed, of which two have not received the combination of multistrain bacteria [23, 34] and the other one has only received probiotics 3 days before surgery [26]. Possible explanations for these controversies may be found in single formulation, small sample size, and short time administration. However, Komatsu et al. [24] found that microbial imbalance could be improved by perioperative probiotics treatment. Similarly, Consoli et al. [23] concluded that probiotic treatment with *Saccharomyces boulardii* downregulates both pro- and anti-inflammatory cytokines in the intestinal colonic mucosa. In addition, a RCT discussed the effects of 12 weeks of probiotics (*Lacidofil*) administration on the quality of life in colorectal cancer patients [45]. The results showed a significantly improved colorectal cancer-related quality of life (FACT) and promising Patient Health-9 (PHQ-9) scores. Furthermore, some studies indicated that probiotics have the potential to prevent tumors, which include those of colorectal cancer [18, 19]; the potential mechanisms may be related to elevation of immune response, increase of short-chain fatty acid production, and reduction of intestinal inflammation (as well as of the mutagenic, carcinogenic, and genotoxic compounds) [46]. Ultimately, probiotics have the potential attractive prospects for use in colorectal surgery.

In conclusion, the combination of multistrain probiotic bacteria (at least three) prevents infectious complications following colorectal surgery, but the efficacy of one or two strains of probiotics remains undetermined.

## Supplementary Material

FIGURE S1: Analysis of subgroups by treatment duration in total infectious. FIGURE S2: Analysis of subgroups by treatment duration in incision infection.

## Figures and Tables

**Table 1:** Characteristics of included studies.

Study	Participants T/C	Age (years) (Mean ± SD)		Intervention		Duration of treatment	Probiotic bacteria	Jadad score
		T	C	T	C			
Consoli et al. [23]	15/18	51.5 ± 13.9	54.5 ± 16.5	Probiotics + antibiotics + MBP	Placebo + antibiotics + MBP	9 d preoperatively	*S. boulardii*	5
Kotzampassi et al. [20]	84/80	65.9 ± 11.5	66.4 ± 11.9	Probiotics + MBP	Placebo + MBP	1 d preoperatively and 15 d postoperatively	*L. acidophilus* *L. plantarum* *B. lactis* *S. boulardii*	4
Komatsu et al. [24]	168/194	66.7 ± 11.6	67.7 ± 10.7	Synbiotics + antibiotics + MBP	Placebo + antibiotics + MBP	7–11 d preoperatively and 2–7 d postoperatively	*L. casei* *B. breve*	3
Sadahiro et al. [25]	100/95	67 ± 9	66 ± 12	Probiotics + antibiotics + MBP	Placebo + antibiotics + MBP	7 d preoperatively and 6 d postoperatively	*B. bifidum*	4
Liu et al. [21]	75/75	62.3 ± 12.4	66.1 ± 11.0	Probiotics + antibiotics + MBP	Placebo + antibiotics + MBP	6 d preoperatively and 10 d postoperatively	*L. plantarum* *L. acidophilus* *B. longum*	5
Zhang et al. [22]	30/30	67.5 ± 10.5	61.5 ± 9.0	Probiotics + antibiotics + MBP	Placebo + antibiotics + MBP	3 d preoperatively	*B. longum* *L. acidophilus* *E. faecalis*	3
Liu et al. [32]	50/50	65.3 ± 11.0	65.7 ± 9.9	Probiotics + antibiotics + MBP	Placebo + antibiotics + MBP	6 d preoperatively and 10 d postoperatively	*L. plantarum* *L. acidophilus* *B. longum*	5
Horvat et al. [26]	20/20	63.0 ± 11.0	65.0 ± 6.5	Synbiotics + antibiotics + MBP	Placebo + antibiotics + MBP	3 d preoperatively	*P. pentosaceus* *L. mesenteroides* *L. paracasei* *L. plantarum*	5
Reddy et al. [27]	20/22	68.4 ± 2.9	69.8 ± 7.0	Synbiotics + antibiotics + MBP	Placebo + antibiotics + MBP	No data	*L. acidophilus* *L. bulgaricus* *B. lactis* *S. thermophilus*	3

MBP: mechanical bowel preparation; L.: *Lactobacillus*; B.: *Bifidobacterium*; P.: *Pediacoccus*; E.: *Enterococcu*s; S.: *Streptococcus.*

**Table 2:** Summary estimates and 95%CIs for total effects of the probiotics.

Outcomes	Number of studies	Case		OR (95%CIs)	*Z*-test (*p* value)	*χ* ^2^	HG^∗^*p* value
		Probiotics	Placebo				
Total infections	5	397	417	0.59 [0.43, 0.83]	0.002	7.11	0.13
Surgical site infection (SSI)		805	850	0.67 [0.49, 0.93]	0.02	8.53	0.67
Incision infection	7	472	491	0.61 [0.41, 0.91]	0.02	4.59	0.60
Organ/space SSI	4	333	359	0.82 [0.47, 1.42]	0.48	3.15	0.53
Nonsurgical site infection (NSSI)		592	587	0.36 [0.23, 0.57]	0.00001	6.26	0.79
Urinary tract infection	3	174	173	0.39 [0.16, 0.96]	0.04	2.06	0.36
Pneumonia	4	209	207	0.25 [0.11, 0.60]	0.002	0.47	0.92
Bacteremia	4	209	207	0.44 [0.23, 0.85]	0.01	2.59	0.46
Bacterial translocation	2	95	97	0.13 [0.01, 1.48]	0.10	6.28	0.01^†^
Anastomotic leakage	4	382	399	0.80 [0.28, 2.48]	0.70	7.08	0.07^‡^

^∗^HG: heterogeneity, *χ*^2^ test with a *p*  value < 0.10 indicated significant heterogeneity across studies; ^†,‡^there was obvious statistical heterogeneity, but no observed clinical heterogeneity, and a random effects model was adopted.

**Table 3:** Summary estimates and 95%CIs for effects of different probiotics formulation.

Outcomes	Number of studies	Case		OR (95%CIs)	*Z*-test (*p* value)	*χ* ^2^	HG^∗^*p* value
		Probiotics	Placebo				
Multistrain probiotic combination							
Total infections	2	114	110	0.30 [0.15, 0.61]	0.0009	0.24	0.62
Surgical site infection (SSI)	—	254	252	0.48 [0.25, 0.89]	0.02	4.12	0.66
Incision infection	5	204	202	0.38 [0.19, 0.76]	0.006	1.10	0.89
Organ/space SSI	2	50	50	2.43 [0.34, 17.15]	0.37	0.04	0.84
Nonsurgical site infection (NSSI)	—	577	569	0.36 [0.23, 0.56]	<0.00001	5.35	0.80
Urinary tract infection	2	159	155	0.34 [0.13, 0.90]	0.03	1.46	0.23
Pneumonia	4	209	207	0.27 [0.11, 0.63]	0.003	0.37	0.95
Bacteremia	4	209	207	0.44 [0.23, 0.85]	0.01	2.59	0.46
Bacterial translocation	2	95	97	0.28 [0.08, 1.01]	0.05	3.03	0.08
Anastomotic leakage	2	114	110	0.14 [0.02, 0.81]	0.03	0.04	0.83
Non-multistrain combination							
Total infections	3	283	307	0.74 [0.50, 1.09]	0.13	2.05	0.36
Surgical site infection (SSI)	—	551	596	0.77 [0.52, 1.12]	0.17	2.60	0.63
Incision infection	2	268	289	0.79 [0.48, 1.31]	0.36	0.76	0.38
Organ/space SSI	3	283	307	0.73 [0.41, 1.31]	0.29	1.86	0.40
Urinary tract infection	1	15	18	1.21 [0.07. 21.22]	0.89	—	—
Anastomotic leakage	2	268	289	1.49 [0.78, 2.86]	0.23	0.85	0.36

^∗^HG: heterogeneity, *χ*^2^ test with a *p*  value < 0.10 indicated significant heterogeneity across studies.
