# Supplementary figures and images for: Probiotics Reduce Postoperative Infections in Patients Undergoing Colorectal Surgery: A Systematic Review and Meta-Analysis
Source: Gastroenterol Res Pract. 2017 Apr 6;2017:6029075. doi: 10.1155/2017/6029075 (PMC5397731; doi:10.1155/2017/6029075)

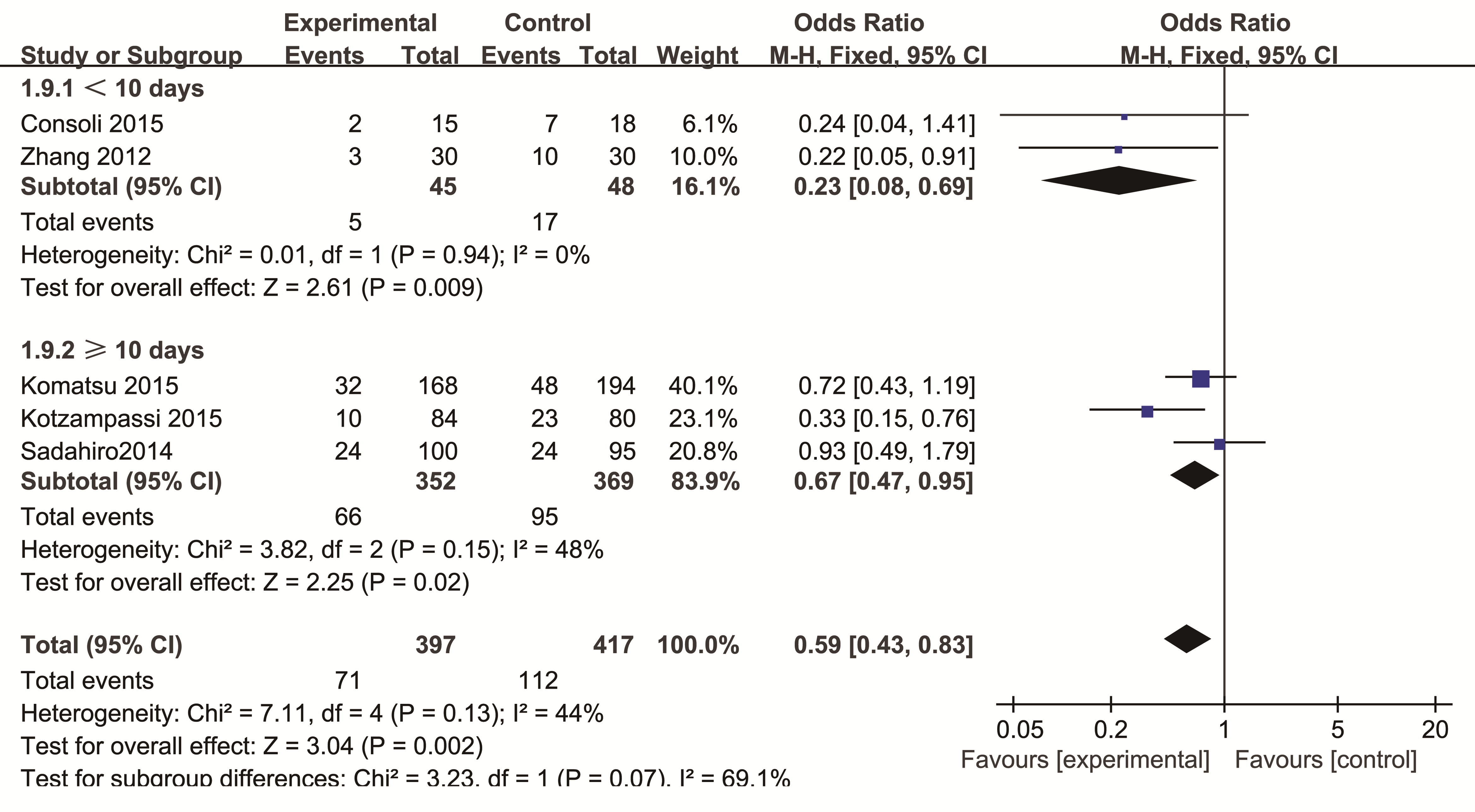


**FIGURE S1:** Analysis of subgroups by treatment duration in total infectious.

Supplement: Supplementary file 1 — FIGURE S1: Analysis of subgroups by treatment duration in total infectious. FIGURE S2: Analysis of subgroups by treatment duration in incision infection. [file 6029075.f1.doc]

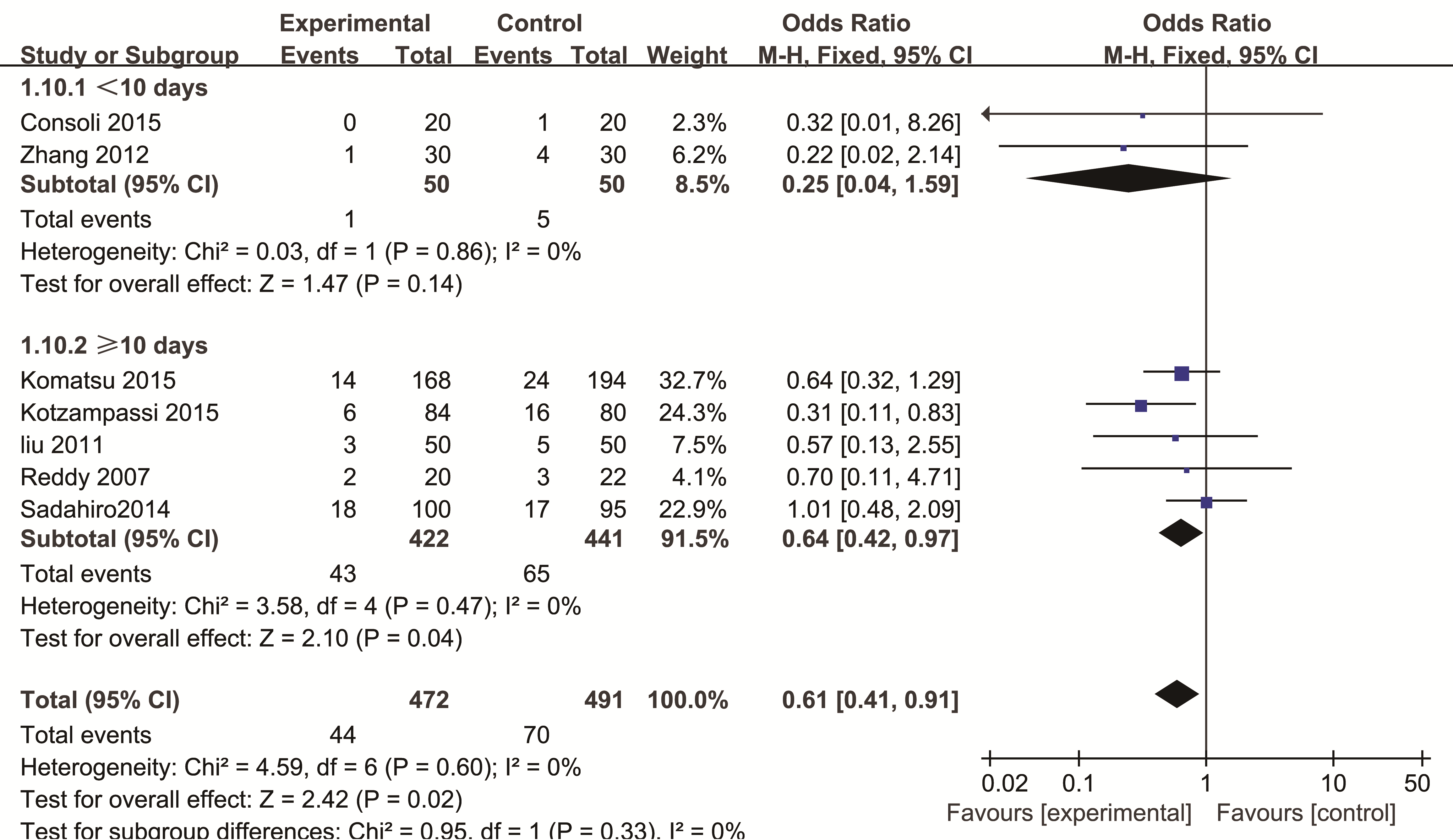


**FIGURE S2:** Analysis of subgroups by treatment duration in incision infection.

Supplement: Supplementary file 3 [file 6029075.f3.doc]
